# Genetic Modification of Cancer Cells Using Non-Viral, Episomal S/MAR Vectors for *In Vivo* Tumour Modelling

**DOI:** 10.1371/journal.pone.0047920

**Published:** 2012-10-26

**Authors:** Orestis Argyros, Suet Ping Wong, Kate Gowers, Richard Paul Harbottle

**Affiliations:** Gene Therapy Research Group, Section of Molecular Medicine, National Heart and Lung Institute, Imperial College London, London, United Kingdom; Enzo Life Sciences, Inc., United States of America

## Abstract

The development of genetically marked animal tumour xenografts is an area of ongoing research to enable easier and more reliable testing of cancer therapies. Genetically marked tumour models have a number of advantages over conventional tumour models, including the easy longitudinal monitoring of therapies and the reduced number of animals needed for trials. Several different methods have been used in previous studies to mark tumours genetically, however all have limitations, such as genotoxicity and other artifacts related to the usage of integrating viral vectors. Recently, we have generated an episomally maintained plasmid DNA (pDNA) expression system based on Scaffold/Matrix Attachment Region (S/MAR), which permits long-term luciferase transgene expression in the mouse liver. Here we describe a further usage of this pDNA vector with the human Ubiquitin C promoter to create stably transfected human hepatoma (Huh7) and human Pancreatic Carcinoma (MIA-PaCa2) cell lines, which were delivered into “immune deficient” mice and monitored longitudinally over time using a bioluminometer. Both cell lines revealed sustained episomal long-term luciferase expression and formation of a tumour showing the pathological characteristics of hepatocellular carcinoma (HCC) and pancreatic carcinoma (PaCa), respectively. This is the first demonstration that a pDNA vector can confer sustained episomal luciferase transgene expression in various mouse tumour models and can thus be readily utilised to follow tumour formation without interfering with the cellular genome.

## Introduction

Cancer represents one of the greatest health risks worldwide. Consequently, there is a growing need for developing novel therapeutics and new advances in animal tumour modelling. However, despite much progress in this field, the development of clinically relevant animal models that permit rapid and sensitive monitoring of early tumour growth and subsequent metastasis remains an on-going challenge [1].

Many conventional animal tumour models used in the development of anticancer treatments involve injection of human tumour cells into immunocompromised mice [2], [3] followed by standard calliper measurements to assess tumour size, usually as an end-point measurement, after the animal has been sacrificed. These models are fairly limited and research has been on-going to develop a genetically marked tumour that would enable non-invasive monitoring of the tumour parameters by *in vivo* imaging based on light emission from luciferase-expressing cells or fluorescence from GFP-expressing cells [1]. The use of genetically marked tumour cells in an animal cancer model has a number of advantages. Primarily, it allows one to monitor the efficacy of therapeutic interventions such as drug, gene or cell therapies more easily than with conventional models. It facilitates tracking of tumour parameters, such as size and development, as well as enables highly sensitive visualisation of early metastasis and the evaluation of minimal residual disease after therapy [4]. It also permits the use of sequential measurements to follow tumour size during treatment so that longitudinal studies can be performed to analyse the effects of therapies over time giving more reliable information and reducing the number of experimental animals [5].

In past studies, a variety of different methods have been employed to endow tumour cells with detectable markers [1], [4], [6], [7], [8], [9]. The most effective method for delivering genes to cells is the use of vectors derived from modified viruses [10]. However, despite the advantages of this gene delivery system there are also significant limitations, mainly related to integration of the vector into the cell genome, the potential immunogenicity of viral encoding genes as well as loss of long-term expression of the reporter gene. It would be of great interest, therefore, to develop a non-viral gene delivery system that can mediate prolonged reporter gene expression in an animal tumour model. An effective way to achieve this goal is to use a plasmid DNA (pDNA) expression system which can be maintained as a functional, episomal entity once it has been delivered to cells of the tumour model and provide them with good detectable levels of marker gene expression throughout their lifetime [11].

Previous *in vivo* studies involving pDNA vectors have shown that viral promoters, such as the cytomegalovirus (CMV) promoter is able to provide the highest levels of transgene expression initially [12], [13] but is followed with a subsequent decline in expression within two months [14]. This decline in expression is promoter-dependent and likely to be the result of transcriptional silencing of the promoter [15]. Indeed, CpG methylation of the CMV promoter in various plasmid vectors has been found to have a negative effect on transgene expression both *in vitro* and *in vivo* [11], [16], [17].

Recently, we and others have shown that a pDNA vector comprising a combination of a mammalian, tissue-specific promoter with a nuclear scaffold/matrix attachment region (S/MAR) element can promote long-term episomal expression *in vitro* and *in vivo* [11], [18], [19], [20], [21]. The S/MAR element provides a specific association of the vector with the nuclear matrix via scaffold attachment factor-A (SAF-A), tethering the vector to the chromosome scaffold during mitosis and bringing the plasmid into close contact with the cell’s replication machinery, therefore creating mitotic stability and maintaining the plasmid as an epigenetic entity through hundreds of cell divisions [22], [23], [24], [25], [26]. The S/MAR element has been shown to have a protective effect on methylation-sensitive sites in the α1-antitrypsin (AAT) liver-specific promoter [11], but has no such effect on the CMV promoter, highlighting that a mammalian rather than a viral promoter is more suitable for long-term transgene expression with this vector.

An S/MAR-containing plasmid has been developed for application to the liver by the utilisation of a liver-specific promoter, AAT, and has been shown to persist and express the luciferase transgene episomally over 6 months in hepatocytes [11]. Given the long-term expression of these episomally maintained plasmids, an S/MAR based vector in combination with a mammalian promoter would appear to be ideal for use as a genetic marker of tumour cells.

Plasmids containing an S/MAR sequence and a CMV promoter have previously been successfully transfected into CHO [18], [23], [25], HaCat [23], HeLa [27], K562 leukaemia cells, U251 glioma [20] and primary fibroblast [28] and have been shown to replicate and to be maintained as extra-chromosomal episomes.

The work described here shows, for the first time, the use of an episomally maintained, pUbC-S/MAR plasmid, mediating persistent luciferase transgene expression to generate genetically labelled tumour cell lines which give rise to different cancers when applied *in vivo*. The cell lines used are a human hepatocellular carcinoma cell-line Huh7, which is derived from a patient with hepatocellular carcinoma and a human pancreatic carcinoma cell-line, MIA-PaCa2.

## Results

### Generation of Stably Transfected Tumour Cell Lines using pUbC-S/MAR Plasmid

**Figure 1:**
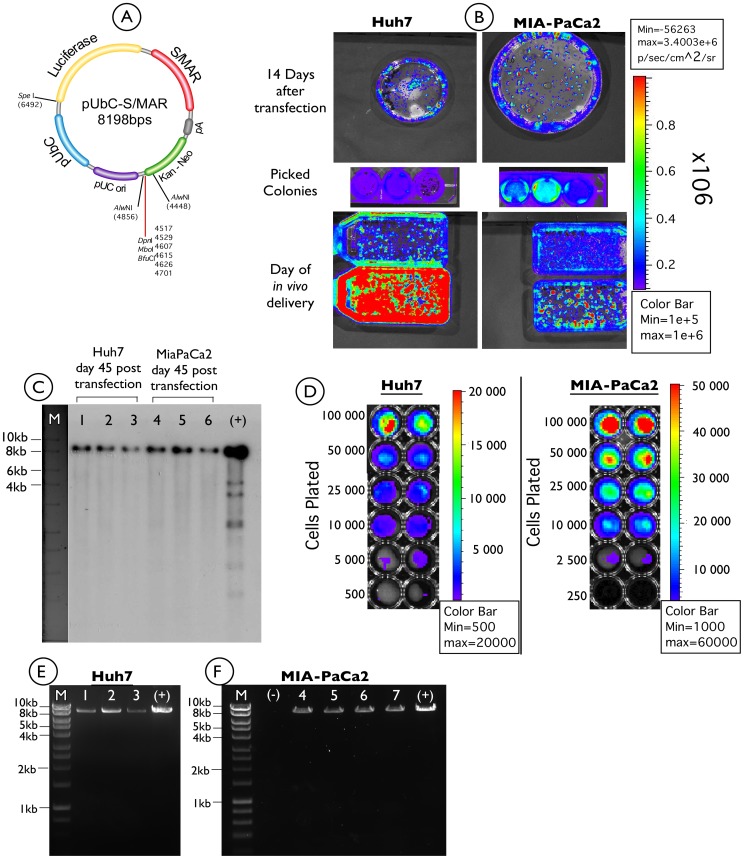
Analysis of luciferase expression from pUbC-S/MAR plasmid in stably transfected Huh7 and MIA-PaCa2 tumour cells. A) The pUbC-S/MAR plasmid used in this study, in which luciferase expression is driven by the human UbC promoter. B) Huh7, and MIA-PaCa2 cells were transfected with pUbC-S/MAR and grown under selection with G418 for about two weeks. Three single colonies were isolated and expanded out of selection with regular imaging using a Xenogen bioimager. C) Southern blot of total DNA isolated from three individual colonies for each cell line at 45 days post transfection. Lanes 1–3: Huh7 isolated colonies; Lanes 4–6 MIA-PaCa2 isolated colonies; (+): Positive control, 10 ng of bacterial pUbC-S/MAR plasmid. D) luciferase bioluminescence assay (in duplicate) on increasing amounts of Huh7 and MIA-PaCa2 cells, showing limits of signal (luciferase) detection, *in vitro*. E–F) Plasmid rescue experiments of three *E.Coli* colonies for Huh7 (lanes 1–3) and four colonies for MIA-PaCa2 cell lines (lanes 4–7), showing identical restriction pattern with pure pUbC-S/MAR plasmid (+), following restriction digest with *Spe*I enzyme. (−) negative control (no DNA); M: 1-kbp ladder (Hyperladder I, Bioline).

Based on previous *in vitro* studies using S/MAR vectors, we aimed to apply this experience to establish a number of different stably transfected tumour cell lines for the generation of different tumour models, which can be monitored by *in vivo* bioluminescence imaging techniques. A plasmid containing an S/MAR element in combination with the mammalian UbC promoter (pUbC-S/MAR), driving a luciferase transgene was used in this study (figure 1A). The ubiquitous UbC promoter was applied so that the same vector could be used to control the luciferase transgene in different cell lines.

The MIA-PaCa2 and Huh7 cells were transfected with the pUbC-S/MAR vector and grown for two weeks in the presence of G418 (1 mg/mL). After this time cells formed distinct colonies and luciferase expression was verified using a bioluminescent imager (figure 1B, top panel). Three individual transgene expressing colonies for each of the cell lines (Huh7 and MIA-PaCa2) were isolated and subsequently cultured without antibiotic selection (figure 1B, middle panel). Expression of the luciferase transgene was demonstrated in both cell lines indicating successful stable transfection with the pUbC-S/MAR plasmid. The cells were further cultured in the absence of selection pressure for another month (figure 1B bottom panel). At 45 days post transfection genomic DNA was extracted from all three colonies of each cell line to confirm episomal maintenance of the pDNA. A Southern blot was performed (figure 1C) which in every case showed a single band of the exact size of pUbC-S/MAR (8198 bps) for each of the colonies of both cell lines. Finally we performed luciferase bioluminescence assays on increasing amounts of cells, in order to provide direct quantitative results of gene expression for comparison. Results are shown in figure 1D, where the limit of signal detection was between 500–5000 cells for Huh7 cells and between 250–2500 cells for MIA-PaCa2.

Further evidence for episomal maintenance was provided by plasmid rescue of pUbC-S/MAR from kanamycin resistant *E. coli* bacteria after transformation with total DNA from each of the colonies of the two cell lines. In this case only intact free plasmid DNA would produce bacterial colonies on plates containing kanamycin that is the resistance marker present on the pUbC-S/MAR plasmid. The restriction patterns of the pDNA of selected colonies were consistent with unmodified non-integrated plasmid constructs for both the Huh7 and the MIA-PaCa2 cell lines (figure 1E and 1F).

### Stably Transfected Huh7 and MIA-PaCa2 Cell Lines form Tumours *in vivo* While Maintaining High Levels of Transgene Expression 35 Days Post Injection

**Figure 2:**
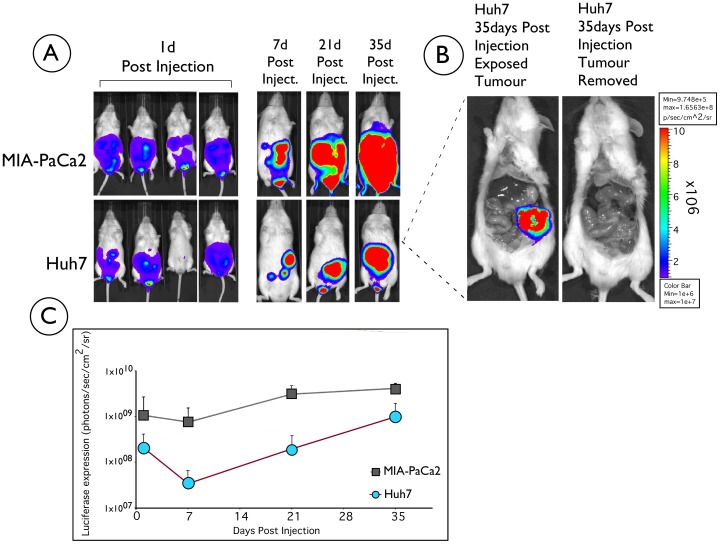
Longitudinal analysis of luciferase expression of pUbC-S/MAR in Huh7 and MIA-PaCa2 cells injected into NOD/SCID mice. A) A group of four mice for each cell line was injected intraperitoneally with 3×10^6^ Huh7 or MIA-PaCa2 cells stably transfected with pUbC-S/MAR and visualised over time (from day one after injection) for bioluminescence using a Xenogen bioimager, following intraperitoneal injections of 15 mg/ml D-luciferin, with one minute acquisition time. One representative mouse for each cell line is shown at days 7, 21 and 35. B) At 35 days post-injection the Huh7 and MIA-PaCa2 injected mice were killed and dissected to look for evidence of tumour growth. No growth was obvious from external examination of the animal, but on opening it up a mass was observed in the peritoneal cavity (Huh7 treated mouse shown as an example). The animal was imaged for bioluminescence before and after tumour removal. The intensity of luciferase expression is shown on the mouse: red represents high expression, violet represents low expression. The colour bar illustrates relative signal intensity. (C) Graphical illustration of the long-term luciferase expression from NOD-SCID mice injected with either Huh7 or MIA-PaCa2 stable cell lines (n = 3 for Huh7 and n = 4 for MIA-PaCa2). Luciferase quantitation is expressed, as photons/sec/cm^2^/sr and plotted (+/− SD). Background level of light emission on non-treated animals is 5×10^5^ photons/sec/cm^2^/sr.

Cells from each of the two generated stably transfected cell lines (MIA-PaCa2 and Huh7) were separately administered by intraperitoneal injection to groups of mice (n = 4). Mice were imaged 24 hours post injection and luciferase expression was observed in both groups of injected mice (Figure 2A). We noticed that all four mice injected with MIA-PaCa2 cells expressed luciferase throughout the monitoring period and formed tumors, whereas three out of four mice injected with Huh7 cells expressed luciferase and one mouse had no initial luciferase expression, probably due to the inability of the cells to establish themselves in the new environment, although this remains unclear. Nevertheless, the luciferase expression was monitored in both groups of mice for a total period of 35 days with weekly bioimaging. Bioluminescent imaging photos of a representative mouse over time for each cell line is shown in Figure 2A. The level of expression increased sharply 21 days post cell delivery (Figure 2C). No luciferase expression was detected in control untreated animals (data not shown), where the background level of light emission was 5×10^5^ photons/sec/cm^2^/sr.

Given the increase in luciferase transgene expression at 35 days post-administration of the cells, we were confident that a tumour derived from the injected cells had formed. The mice were therefore sacrificed at 35 days and dissected to look for evidence of tumour formation. While externally there was no noticeable growth, a large mass was observed in the peritoneal cavity once the animal was dissected. A representative photo of the tumour mass from an animal treated with Huh7 cells is shown in Figure 2B. Imaging of the mice before and after the removal of the tumour confirmed that luciferase transgene expression was localised to the tumour mass (Figure 2B).

### Histological Analysis of the Formed Tumours

**Figure 3:**
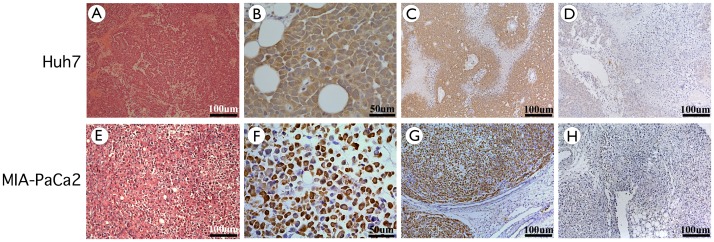
Histochemistry and Immunohistochemistry of tumour sections at day 35 post delivery, showing the formation of a hepatocellular carcinoma-like tumour and a pancreatic carcinoma tumour, to which luciferase expression localises. Sections from different parts of the two tumours were cut and stained with haematoxylin and eosin for histological analysis of the tumours. A–D) Sections from Huh7 injected mice. Sections have an amorphous structure and were identified as hepatocellular carcinoma (HCC) of varying degrees of differentiation: (A) Moderately differentiated HCC, magnification×10 (B–C) Sections were analysed by immunohistochemistry to show distribution of luciferase expression. Brown staining indicates luciferase positive cells. (B) Positively stained, Magnification×40 (C) Positively stained, Magnification×10 (D) Negative control: no primary antibody added, magnification×10 E–H) Sections from MIA-PaCa2 injected mice. Sections have an amorphous structure and were identified as Pancreatic carcinoma (PaCa) of varying degrees of differentiation. (E) Moderately differentiated PaCa, magnification×10 (F–G) Sections were analysed by immunohistochemistry to show distribution of luciferase expression. Brown staining indicates luciferase positive cells. (F) Positively stained, Magnification×40 (G) Positively stained, Magnification×10 (H) Negative control: no primary antibody added, magnification×10.

Haematoxylin and eosin stained tissue sections were performed to identify tumour histology derived from each cell line. Figure 3 shows histology sections of tumours formed from Huh7 cells. Histology confirms that the tumour is a hepatocellular carcinoma (HCC) with varying degrees of differentiation (Figure 3A–D). The tumour is composed of polygonal cells distributed in loose sheets and pseudoglandular patterns. The nuclei were moderately pleomorphic, vesicular and contain a nucleolus. A few isolated mitotic figures were also noted. The cytoplasm was eosinophilic and the cell borders were well defined, while the stroma was scanty. Intracellular and extracellular bile droplets were not seen in the tumour and neither was tumour necrosis. The features of the tumour were confirmed by an independent histopathologist to be consistent with a Grade II HCC (modified Edmonson and Steiner’s grading system).

In addition luciferase immunohistochemical analysis of tumour sections (Figure 3B and 3C) showed all hepatocyte-like cells derived from the injected cells to be expressing luciferase. Unstained areas are believed to be either necrotic tissue or cells recruited to the tumour, which has not yet been confirmed experimentally and is currently under investigation.

Similarly, haemotoxylin and eosin stained tissue sections were obtained for the tumours formed in mice after injection of MIA-PaCa2 cells (Figure 3E–H). In this case, the histological sections revealed that the formed tumour cells had permeated between the normal pancreatic acini at the periphery of the tumour. The tumour cells were described to be distributed in solid sheets with no evidence of glandular differentiation and have a moderate amount of cytoplasm with well-defined cell borders. The nuclei were atypical and hyperchromatic while nucleoli were inconspicuous. Mitotic figures were rare. The majority of the tumour cells contained a pale intracytoplasmic vesicle pushing the nucleus to the periphery imparting a signet ring appearance. Extracellular mucus and stromal fibrosis were not seen. An independent histopathologist confirmed all the features of the tumour were consistent with a signet ring carcinoma of pancreas.

### The pUbC-S/MAR Plasmid is Episomally Maintained and Expressed in the Resulting Tumour Tissues

To provide physical evidence of the molecular nature of the pUbC-S/MAR plasmid in the HCC and pancreatic tumours, we performed Southern blot analysis on total DNA isolated from two different sites of the same tumour at 35 days post delivery of either the Huh7 or the MIA-PaCa2 cell line.

**Figure 4:**
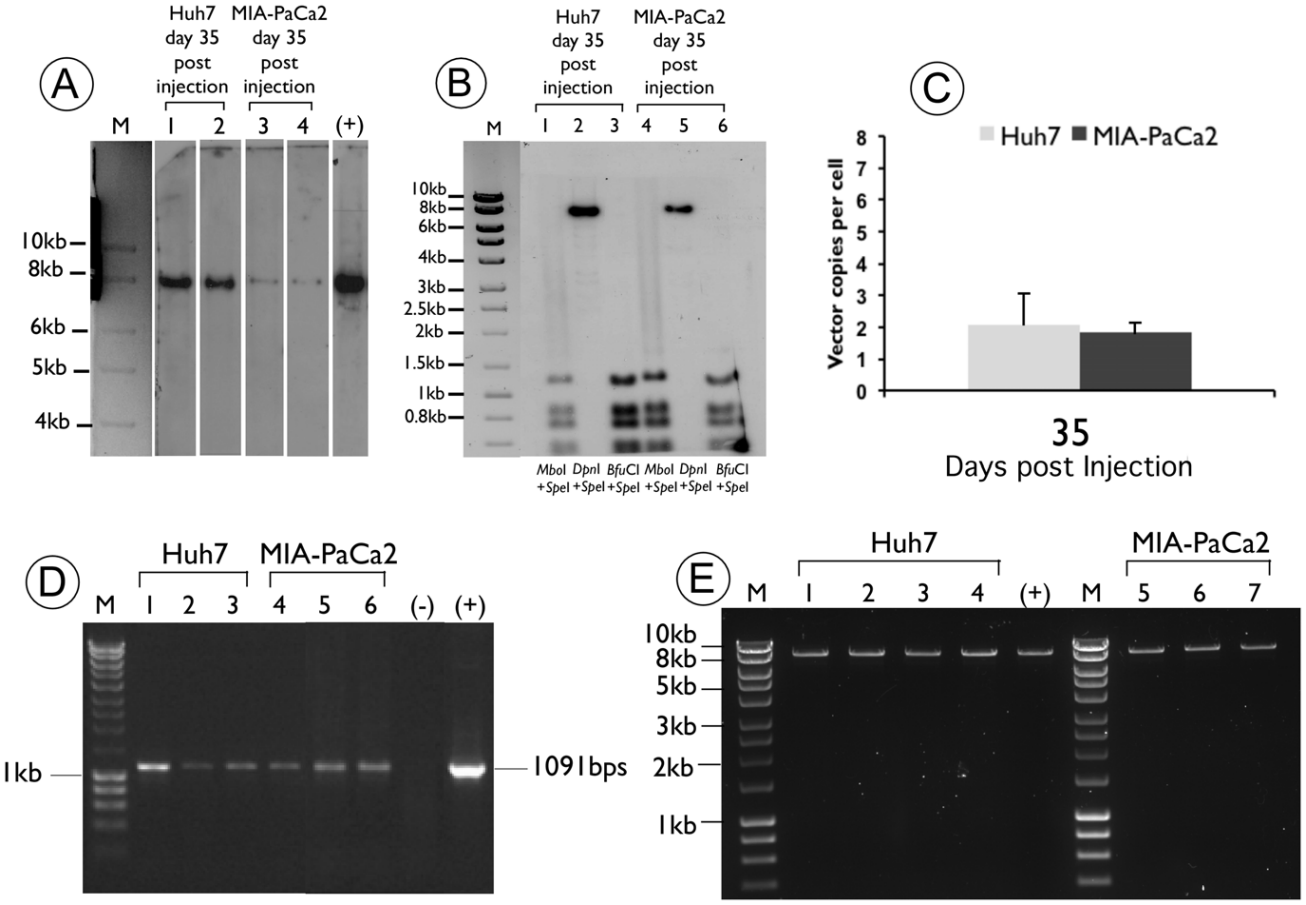
Molecular analysis of DNA isolated from tumour tissues at day 35 post delivery, from Huh7 and MIA-PaCa2 injected NOD/SCID mice. (A) Southern blot analysis of pDNA isolated from two different regions of tumour tissue from NOD/SCID mice, 35days post-delivery of Huh7 and MIA-PaCa2 stable cell lines, performed as described in materials and methods. A representative hybridization pattern of pDNA isolated from one animal of each tumour is shown. Detection of indicator plasmid by M: 1-kbp ladder (Hyperladder I, Bioline); lane 1: pUbC-S/MAR isolated from the tumour tissue formed after Huh7 injection of NOD/SCID mice at 35 days post-injection; lane 2: pUbC-S/MAR isolated from a different region of the tumour tissue formed after Huh7 delivery into NOD/SCID mice at 35 days post-injection; lane 3 pUbC-S/MAR isolated from the tumour tissue formed after MIA-PaCa2 injection of NOD/SCID mice at 35 days post-injection; lane 4: pUbC-S/MAR isolated from a different region of the tumour tissue formed after MIA-PaCa2 delivery into NOD/SCID mice at 35 days post-injection; (+) positive control: 25 ng of linearized pUbC-S/MAR plasmid. (B) Replication-dependent assay of pUbC-S/MAR plasmid DNA isolated from the tumours of mice at 35 days post-administration. lanes 1–3: Southern blot of total tumour DNA isolated from NOD/SCID mice at 35 days post-delivery with Huh7 stable cell line and double digested with *Spe*I–*Mbo*I (lane 1), *Spe*I–*Dpn*I (lane 2) or *Spe*I–*Bfu*CI (lane 3) enzymes; lanes 7–9: Southern of total tumour DNA isolated from NOD/SCID mice at 35 days post-delivery with MIA-PaCa2 stable cell line and double digested with *Spe*I–*Mbo*I (lane 4), *Spe*I–*Dpn*I (lane 5) or *Spe*I–*Bfu*CI (lane 6) enzymes; M: 1-kbp ladder (Hyperladder I, Bioline UK Ltd., London, UK). (C) Quantitative PCR performed on tumour DNA obtained at day 35 after injection of Huh7 and MIA-PaCa2 cell lines. DNA was extracted from two different sites of each tumour at the end of the experiment and the number of pUbC-S/MAR vector genomes per diploid genome is shown, after normalisation with GAPDH gene, as described in materials and methods. (D) PCR analysis of DNA isolated *in vitro* from the Huh7 (lane 1) and MIA-PaCa2 (lane 4) cells before injection into NOD/SCID mice, and *in vivo* from two different regions of the tumour for each cell line (lanes 2,3 for Huh7 and lanes 5,6 for the MIA-PaCa2 cell lines). Expected PCR product size: 1091 bp. 100 bp DNA ladder (lane M), (+) positive control: pUbC-S/MAR; (-) negative control: PCR mix without DNA. (E) Plasmid rescue experiments of four *E.Coli* colonies for Huh7 (lanes 1–4) and three colonies for MIA-PaCa2 cell lines (lanes 5–7), showing identical restriction pattern with pure pUbC-S/MAR plasmid (+), following restriction digest with *Spe*I enzyme. M: 1-kbp ladder (Hyperladder I, Bioline).

A representative blot is shown in Figure 4A where an individual band of the expected size is detected in all lanes. This indicates that the pUbC-S/MAR pDNA remain episomal at 35 days post-delivery. Further evidence for episomal maintenance was also provided by plasmid rescue of pUbC-S/MAR plasmid isolated from kanamycin-resistant *E. coli* after transformation with total DNA from the tumour derived from Huh7 or MIA-PaCa2 treated mice. In this case, only intact free pDNA from the isolated tumour sample would produce bacterial colonies on plates containing kanamycin, the resistance marker present on the plasmid. The restriction patterns of the pUbC-S/MAR plasmid were consistent with unmodified non-integrated plasmid constructs (shown in Figure 4E).

In addition, we performed a replication dependent restriction assay to show pDNA replication. Total tumour DNA from two different areas of either the HCC or the PaCa tumour, were isolated from animal groups treated with pUbC-S/MAR plasmid at the end of the 35 days experiment and was digested with *Spe*I, a single cutter, to linearize the plasmid, before further digestion overnight with the methylation-sensitive enzymes *Dpn*I, *Mbo*I or *Bfu*CI. All three enzymes recognize the same sequence (GATC). *Dpn*I requires methylation of the target DNA by bacterial cells for digestion, while *Mbo*I restriction is dependent on mammalian DNA methylation and *Bfu*Cl cuts regardless of methylation status and does not distinguish the source of methylation.

The restriction digestion fragments were separated on a 0.8% agarose gel, then blotted and probed with a 408-bp fragment from the kanamycin resistance gene. The Southern blot analysis (Figure 4B) serves to compare the digestion pattern of pUbC-S/MAR in tumour DNA isolated from the Huh7 treated animal group (Figure 4B lanes 1–3) with that from the MIA-PaCa2 group (Figure 4B lanes 4–6).

A loss of bacterial methylation of the pUbC-S/MAR plasmid is found in DNA isolated from both tumours when these are digested with *Spe*I/*Mbo*I or *Spe*I/*Dpn*I, respectively (lanes 1,2,4,5). Successful digestion by *Mbo*I is indicated by conversion of the linear *Spe*I band to digestion fragments (lane 1 for Huh7 and lane 4 for MIA-PaCa2), and lack of digestion by *Dpn*I leaves the single *Spe*I linearized band intact (lane 2 for Huh7 and lane 5 for MIA-PaCa2). As *Mbo*I only cuts mammalian-derived DNA, while *Dpn*I requires bacterial methylation for digestion, this indicates that pUbC-S/MAR plasmid has replicated in tumor cells of both the HCC and PaCa. Finally, digestion with *Spe*I-*Bfu*CI is not blocked by any kind of methylation and serves as a positive control for plasmid digestion (lanes 3 and 6). These data indicate that the S/MAR-harbouring plasmid pUbC-S/MAR is able to replicate *in vivo* after delivery of stably transfected cell lines, similar to studies *in vitro* in which S/MAR-endowed pDNA is able to achieve mitotic stability and replication [26]. The correct size of the restriction digestion bands suggests mitotic stability without gross rearrangements of the replicating plasmid.

Quantitative PCR was performed at the termination of the experiment (at 35 days post delivery) to compare the relative copy number of plasmid molecules in the Huh7 and MIA-PaCa2 treated groups. The results are shown in Figure 4C, where in both cases the plasmid copy number is calculated to be about one to three vector copies/cell, consistent with previous reports demonstrating a relatively low copy number of S/MAR vector of less than 10 copies/cell [18], [25], [29].

In addition, maintenance of the transgenic marker gene was also shown by PCR analysis on DNA isolated from “cultured” MIA-PaCa2 and Huh7 cells (Figure 4D, lanes 1 and 4) and from two different sites of tumour tissue at 35 days after delivery (Figure 4D, lanes 2,3 for Huh7 and lanes 5,6 for MIA-PaCa2). A PCR product of the expected size, 1091 bps, was generated from DNA of all sources indicating that the pUbC-S/MAR plasmid was maintained in both the Huh7 and MIA-PaCa2 cells and throughout the tumour derived from these cells after injection into NOD-SCID mice. This verifies the presence of the pUbC-S/MAR plasmid both *in vitro* and *in vivo.*

## Discussion

This work represents the development of murine tumour models derived from two different cell lines. Significantly, this study shows for the first time the establishment of genetically marked murine models of pancreatic and hepatocellular carcinomas using a non-viral episomal plasmid vector. Both HCC and PaCa have high incidences; HCC is the fifth most common form of cancer in the world and accounts for 80–90% of primary liver cancer [30] while there are around 42470 individuals diagnosed with pancreatic cancer each year in the United States with less than a 20% one-year survival rate [31].

Given the prevalence of these diseases, it is vital that an effective method be developed to improve the disease detection and prognosis. The generation of an effective genetically marked murine tumour model for HCC and PaCa is an important step in this process as it will enable the effects of potential therapeutics to be more easily and accurately monitored and will therefore enable more reliable data when developing novel anticancer drugs.

Attempts to generate genetically marked tumours previously have had limitations, such as the risk of integration with viral vectors and potential insertional mutagenesis. Furthermore the genotoxicity of viral vectors can considerably alter the characteristics of its recipient cell and subsequent daughter cells. When creating tumour xenografts, the fewer alterations made to the original tumour cells the better the representation of the cancer model. Therefore the development of non-viral vectors in cancer research to minimise these adverse effects are crucial. Our previous work has shown strong sustained episomal luciferase expression *in vivo*, from a pDNA expression system comprising an S/MAR element and a mammalian promoter in the murine liver [11], [20], [21]. Tracking of the luciferase transgene over time in a single animal without the need for sacrificing animals indicates the utility of this vector in genetically marked tumour cells to track the development of a tumour model simply by *in vivo* imaging. As shown here, the S/MAR vector enables stable transfection of cancer cells and subsequent development of HCC and PaCa tumour xenografts. This paper describes the first demonstration of the functional use of an S/MAR vector to stably transfect cancer cells to genetically mark tumours *in vivo*.

Previous studies *in vitro* have shown that S/MAR vectors can replicate episomally irrespective of the promoter used. We confirm and extend this observation using the pUbC-S/MAR vector in Huh7 and MIA-PaCa2 cell lines. We have obtained similar results by using the pEPI-Luc vector - an S/MAR plasmid where luciferase expression is driven by the human CMV promoter (data not shown). However, a previous study to mark tumour cells genetically with a luciferase transgene driven by the CMV promoter [4] has shown the limitations of this promoter for long-term transgene expression since the CMV promoter is readily inactivated by several host mechanisms such as CpG methylation [11], [14], [15], [16], [17]. This limitation has been overcome by our study, which demonstrates a sustained expression from the mammalian UbC promoter in combination with an S/MAR element. Differential establishment of cells can account for differences in luciferase expression seen between animals in each group following administration.

Histopathology analysis of the tumours showed the typical tissue morphology expected of PaCa and HCC (Figure 3) and the immunohistochemical analysis showed all tumour cells derived from those injected into the mouse to be luciferase positive (Figure 3). Given this and the long-term transgene expression achieved for 35 days post-injection where a steep increase of expression is observed after 21 days (Figure 2C), this S/MAR vector seems to be ideally suited for use in cancer cell lines to generate a genetically marked murine model of this disease. The maintenance of transgene expression for 35 days is significant and given past *in vivo* investigations with a similar vector [11], we assume that expression should persist for several more months. Due to associated animal welfare issues, extending the time period for this study of tumour models is not feasible and therefore the time period of the study presented here is likely to be fairly representative of most animal tumour model studies. In addition to maintaining long-term reporter gene expression, pUbC-S/MAR was shown to be episomally retained and capable of replication *in vitro* and *in vivo* after multiple rounds of cell division confirming previous findings [18], [19], [23], [25], [27], [28]. Furthermore this paper shows for the first time the ability of an S/MAR vector to replicate episomally in injected tumour cells *in vivo*.

In conclusion, the work presented here highlights the suitability of pUbC-S/MAR pDNA vector as a genetic marker of murine tumour models. In addition to being non-viral in design it is able to facilitate episomal maintenance and long-term transgene expression. Furthermore, our model illustrates the ease and speed in which a vector can be used to stably transfect tumor cells for generating genetically marked tumor models for the development and monitoring of potential therapies in approximately one month. This work can have important applications in the field of anti-cancer drug development for treating HCC or PaCa but also for other cancers, provided that stable cell lines can be generated as shown in the current work.

## Materials and Methods

### Ethics Statement

Animal studies were carried out in accordance with UK Research Councils’ and Medical Research Charities’ guidelines on Responsibility in the Use of Animals in Bioscience Research, under a UK Home Office license (PPL# 70/6906; Title: Development of gene transfer vectors as therapeutics and biosensors).

### Plasmid Vectors

The pUbC-S/MAR (kindly provided by Dr Carsten Rudolph, University of Munich, Germany) and the pEPI-Luc (kindly provided by Professor Hans J Lipps, University of Witten, Germany) plasmids used are derived from the commercially available plasmid pGFP-C1 (Clontech, Mountain View, CA, USA). The plasmids were amplified in *E.coli* DH10B cells (Invitrogen, Paisley, UK) and purified using a Maxi-prep kit (Qiagen, Crawley, UK). Diagnostic restriction enzymes were used to digest maxi-preps to ensure purification of the correct plasmid DNA.

### Mammalian Cell Culture

Both Huh7 (ATCC number: CCL-185) and MIA-PaCa2 (ATCC number: CRL-1420) tumour cell lines were purchased from ATCC. Tumour cell lines were grown at 37°C in Dulbecco’s modified eagle’s medium (DMEM) (Invitrogen, UK) supplemented with 10% foetal calf serum (FCS) and 1% penicillin/streptomycin. For the generation of stably transfected tumour cell lines, cells were transfected using LipofectAMINE 2000 (Invitrogen, UK) according to the manufacturer’s guidelines. Transfected cells were grown under selection with 1 mg/ml G418 (Sigma, Poole, UK) for approximately two weeks. Single colonies were isolated, removed from selection and grown to expand the population. For bioimaging, D-luciferin (150 µg/ml) (Gold Biotechnology, USA) was diluted in DMEM and added to the cells for 10 minutes before being imaged for bioluminescence using an IVIS Imaging 50 Series (Xenogen, Alameda, CA, USA). The background level of bioluminescence on untreated cells is 1×10^5^ photons/sec/cm^2^/seradian (sr).

### Administration of Cells in NOD-SCID Mice

NOD-SCID (Non-Obese Diabetic/Severe Combined ImmunoDeficient) mice (Harlan Ltd., UK) were injected intraperitoneally with 3×10^6^ Huh7, or MIA-PaCa2 cells suspended in 150 µl PBS. After 24 hours and at regular time intervals after injection, mice were imaged for bioluminescence using the IVIS Imaging 50 Series as described above, using an acquisition time of one minute and a pixel binning of 8. Briefly, the mice were anaesthetised by isoflurane, intraperitoneally injected with 300 µl D-luciferin (15 mg/ml in PBS) and imaged. Data were analysed using LivingImage 2.50 software (Xenogen). The background level of light emission on non-treated animals is 5×10^5^ photons/sec/cm^2^/sr. Animals were maintained and cared for in accordance with institutional and UK guidelines.

### PCR Analysis

DNA was isolated from cells and two different tumour sites using a DNeasy Tissue kit (Qiagen, UK). PCR was conducted in a Primus 96 Plus PCR Thermocycler (MWG AS Biotech, Ebersberg, Germany) with the following primers (Invitrogen): UBC promoter: forward 5′-GAACAGGCGAGGAAAAGTAGTCC-3′; reverse 5′-ACCAGGGCGTATCTCTTCATAGC-3′; product size: 1091 bp. Reactions were set up using 3 mM MgCl_2_ (Invitrogen, UK), 0.2 mM each dNTPs (Invitrogen, UK), 1×PCR buffer (Invitrogen, UK), 0.5 µM of each forward and backward primer (Invitrogen, UK), 100 ng DNA, 0.25 µl Taq (5 U/µl) (Invitrogen, UK) and the final volume adjusted to 50 µl with dH_2_O. Template DNA was initially denatured at 95°C for 5 minutes, followed by 30 cycles of denaturation at 95°C for 45 seconds, annealing at 60°C for 45 seconds and primer extension at 72°C for 1 minute. A final 10-minute incubation at 72°C allowed for complete extension. PCR products were analysed on 0.8% agarose gels.

### Southern Blot Analysis

For DNA analysis, total cellular or tumour DNA (collected from two different tumour sites) was extracted using a DNeasy Tissue kit (Qiagen, UK). The isolated DNA was quantified using a NanoDrop ND1–1000 spectrophotometer (Labtech International Ltd, Ringmer, UK). For Southern analysis, total tumour DNA (15 µg) was digested with the single cutting restriction enzyme (*Spe*I) and separated on 0.8% agarose gels (20 V, 20 mA overnight) and blotted onto nylon membranes (Hybond XL, Amersham plc, Little Chalfont, UK). A 408 bp DNA fragment derived from the restriction digest of a segment of the kanamycin region, which is common to all plasmids, using enzyme *Alw*NI, was labelled with 32P (Rad-Prime labelling kit, Invitrogen, UK) and used as a probe. The hybridization was performed in Church buffer (0.25 M sodium phosphate buffer (pH 7.2), 1 mM EDTA, 1% BSA, 7% SDS) at 65**°**C for 16 h.

For the replication-dependent restriction assay, 15 µg of total tumour DNA was digested with *Spe*I and further digested with *DpnI*, *Mbo*I or *Bfu*CI enzyme overnight. Agarose gel separation and Southern analysis was then performed as mentioned above.

### Plasmid Rescue Experiments

Stbl3 *E. coli* cells (Invitrogen, UK) were transformed by heat-shock, using 15 µg DNA prepared by total cellular and tumour DNA isolation. DNA was concentrated using a Genomic DNA Clean and Concentrator kit (Zymo Research, USA) according to manufacturer’s instructions. Transformed colonies were selected on agar plates containing 30 µg/ml kanamycin. DNA was isolated from individual resistant clones, subjected to restriction analysis (*Spe*I), and analysed by electrophoresis on 0.8% agarose gels.

### Quantitative PCR

Relative amounts of plasmid DNA in tumour samples were calculated by real-time PCR using the Syber Green PCR system (ABI) on an Applied Biosystems 7500 Fast Real-Time PCR System, with 40 cycles per sample. Cycling temperatures were as follows: denaturing 95**°**C, annealing and extension, 60**°**C. OligoPerfect**™** Designer software was used to design oligonucleotide primers (Invitrogen, UK) and luciferase expression was used to determine amounts of S/MAR plasmid. Primers specific for the GAPDH gene (Invitrogen, UK) were used to enable normalisation between the samples through calculating the number of cells used as the input. The following primers were used: Luciferase: Forward: 5′-GGCGCGTTATTTATCGGAGTT- 3′; Reverse: 5′-CCATACTGTTGAGCAATTCACGTT-3′; GAPDH: Forward: 5′-ACCACAGTCCATGCCATCAC-3′; Reverse: 5′-TCCACCCTGTTGCTGTA-3′. Serial dilutions of plasmids containing appropriate sequences to produce a standard amplification curve for quantification and all samples were tested in triplicate.

### Immunohistochemistry

Tumour tissue was fixed in paraformaldehyde and paraffin wax-embedded, before being cut into sections 4 µm in thickness. Sections were taken through histoclear (National Diagnostics, Georgia, USA) and a series of decreasing concentrations of ethanol to dehydrate them. Sections were stained with haematoxylin and eosin to observe tissue morphology. For immunohistochemical analysis of luciferase expression, sections were incubated in 3% hydrogen peroxide to block endogenous peroxidases and rinsed in ethanol solutions of decreasing concentration to rehydrate the sample. Sections were incubated in 0.01 M sodium citrate buffer and treated with avidin and biotin (Vector Laboratories, CA, USA). Sections were blocked in horse serum and then incubated overnight at 4°C with a 1∶50 dilution of rabbit monoclonal anti-luciferase antibody (Santa Cruz Biotechnology, Santa Cruz, USA). The next day sections were incubated with a 1∶1000 dilution of biotin-conjugated horse anti-rabbit immunoglobulin (Vector Labs) followed by addition of the Vectastain ABC complex (Vector Labs) according to the manufacturer’s instructions. Colour was developed by incubation with DAB substrate (Vector Labs) for 5 minutes. Slides were stained with haematoxylin and dehydrated by rinsing in a series of ethanol solutions of increasing concentration, before being mounted and visualised using an LEICA DM4000 B microscope with a LEICA DFC420 camera inverted microscope. Image acquisition and analysis was performed using Leica LAS software, Lite version.

## References

[1] Jenkins DE, Oei Y, Hornig YS, Yu SF, Dusich J, et al (2003) Bioluminescent imaging (BLI) to improve and refine traditional murine models of tumor growth and metastasis. Clinical & experimental metastasis 20: 733–744. 14713107 10.1023/b:clin.0000006815.49932.98

[2] Kerbel RS (1998) What is the optimal rodent model for anti-tumor drug testing?. Cancer metastasis reviews 17: 301–304. 10352884 10.1023/a:1006152915959

[3] Killion JJ, Radinsky R, Fidler IJ (1998) Orthotopic models are necessary to predict therapy of transplantable tumors in mice. Cancer metastasis reviews 17: 279–284. 10352881 10.1023/a:1006140513233

[4] Wetterwald A, van der Pluijm G, Que I, Sijmons B, Buijs J, et al (2002) Optical imaging of cancer metastasis to bone marrow: a mouse model of minimal residual disease. The American journal of pathology. 10.1016/S0002-9440(10)64934-6 PMC1867183 11891210

[5] Stell A, Biserni A, Della Torre S, Rando G, et al (2007) Cancer modeling: modern imaging applications in the generation of novel animal model systems to study cancer progression and therapy. The international journal of biochemistry & cell biology 39: 1288–1296. 17418611 10.1016/j.biocel.2007.02.019

[6] Edinger M, Sweeney TJ, Tucker AA, Olomu AB, Negrin RS, et al (1999) Noninvasive assessment of tumor cell proliferation in animal models. Neoplasia 1: 303–310. 10935484 10.1038/sj.neo.7900048 PMC1508101

[7] Aboody-Guterman KS, Pechan PA, Rainov NG, Sena-Esteves M, Jacobs A, et al (1997) Green fluorescent protein as a reporter for retrovirus and helper virus-free HSV-1 amplicon vector-mediated gene transfer into neural cells in culture and in vivo. Neuroreport 8: 3801–3808. 9427374 10.1097/00001756-199712010-00029

[8] Yang M, Baranov E, Jiang P, Sun FX, Li XM, et al (2000) Whole-body optical imaging of green fluorescent protein-expressing tumors and metastases. Proceedings of the National Academy of Sciences of the United States of America 97: 1206–1211. 10655509 10.1073/pnas.97.3.1206 PMC15570

[9] Dikmen ZG, Gellert GC, Dogan P, Yoon H, Lee YB, et al (2008) In vivo and in vitro effects of a HIF-1alpha inhibitor, RX-0047. Journal of cellular biochemistry 104: 985–994. 18275063 10.1002/jcb.21681 PMC3375689

[10] Dinca EB, Sarkaria JN, Schroeder MA, Carlson BL, Voicu R, et al (2007) Bioluminescence monitoring of intracranial glioblastoma xenograft: response to primary and salvage temozolomide therapy. Journal of neurosurgery 107: 610–616. 17886562 10.3171/JNS-07/09/0610

[11] Argyros O, Wong SP, Niceta M, Waddington SN, Howe SJ, et al (2008) Persistent episomal transgene expression in liver following delivery of a scaffold/matrix attachment region containing non-viral vector. Gene Ther 15: 1593–1605. 18633447 10.1038/gt.2008.113

[12] Wang Y, Sun Z, Peng J, Zhan L (2007) Bioluminescent imaging of hepatocellular carcinoma in live mice. Biotechnology letters 29: 1665–1670. 17609854 10.1007/s10529-007-9452-0

[13] Zeamari S, Rumping G, Floot B, Lyons S, Stewart FA (2004) In vivo bioluminescence imaging of locally disseminated colon carcinoma in rats. British Journal of Cancer 90: 1259–1264. 15026810 10.1038/sj.bjc.6601637 PMC2409642

[14] Wilber A, Frandsen JL, Wangensteen KJ, Ekker SC, Wang X, et al (2005) Dynamic gene expression after systemic delivery of plasmid DNA as determined by in vivo bioluminescence imaging. Hum Gene Ther 16: 1325–1332. 16259566 10.1089/hum.2005.16.1325

[15] Herweijer H, Zhang G, Subbotin VM, Budker V, Williams P, et al (2001) Time course of gene expression after plasmid DNA gene transfer to the liver. The journal of gene medicine 3: 280–291. 11437333 10.1002/jgm.178

[16] Brooks AR, Harkins RN, Wang P, Qian HS, Liu P, et al (2004) Transcriptional silencing is associated with extensive methylation of the CMV promoter following adenoviral gene delivery to muscle. J Gene Med 6: 395–404. 15079814 10.1002/jgm.516

[17] Hong K, Sherley J, Lauffenburger DA (2001) Methylation of episomal plasmids as a barrier to transient gene expression via a synthetic delivery vector. Biomolecular engineering 18: 185–192. 11576873 10.1016/s1389-0344(01)00100-9

[18] Piechaczek C, Fetzer C, Baiker A, Bode J, Lipps HJ (1999) A vector based on the SV40 origin of replication and chromosomal S/MARs replicates episomally in CHO cells. Nucleic Acids Res 27: 426–428. 9862961 10.1093/nar/27.2.426 PMC148196

[19] Jenke AC, Stehle IM, Herrmann F, Eisenberger T, Baiker A, et al (2004) Nuclear scaffold/matrix attached region modules linked to a transcription unit are sufficient for replication and maintenance of a mammalian episome. Proc Natl Acad Sci U S A 101: 11322–11327. 15272077 10.1073/pnas.0401355101 PMC509201

[20] Argyros O, Wong SP, Fedonidis C, Tolmachov O, Waddington SW, et al (2011) Development of S/MAR minicircles for enhanced and persistent transgene expression in the mouse liver. Journal of Molecular Medicince 89: 515–529. 10.1007/s00109-010-0713-3 21301798

[21] Wong SP, Argyros O, Coutelle C, Harbottle RP (2011) Non-viral S/MAR vectors replicate episomally in vivo when provided with a selective advantage. Gene Ther 18: 82–87. 20739959 10.1038/gt.2010.116

[22] Argyros O, Wong SP, Harbottle RP (2011) Non-viral episomal modification of cells using S/MAR elements. Expert Opin. Biol. Ther. 11: 1177–1191. 10.1517/14712598.2011.582035 21548848

[23] Jenke AC, Scinteie MF, Stehle IM, Lipps HJ (2004) Expression of a transgene encoded on a non-viral episomal vector is not subject to epigenetic silencing by cytosine methylation. Mol Biol Rep 31: 85–90. 15293783 10.1023/b:mole.0000031363.35839.46

[24] Lufino MM, Manservigi R, Wade-Martins R (2007) An S/MAR-based infectious episomal genomic DNA expression vector provides long-term regulated functional complementation of LDLR deficiency. Nucleic Acids Res 35: e98. 17675302 10.1093/nar/gkm570 PMC1976449

[25] Stehle IM, Postberg J, Rupprecht S, Cremer T, Jackson DA, et al (2007) Establishment and mitotic stability of an extra-chromosomal mammalian replicon. BMC Cell Biol 8: 33. 17683605 10.1186/1471-2121-8-33 PMC1959191

[26] Jenke BH, Fetzer CP, Stehle IM, Jonsson F, Fackelmayer FO, et al (2002) An episomally replicating vector binds to the nuclear matrix protein SAF-A in vivo. EMBO Rep 3: 349–354. 11897664 10.1093/embo-reports/kvf070 PMC1084058

[27] Schaarschmidt D, Baltin J, Stehle IM, Lipps HJ, Knippers R (2004) An episomal mammalian replicon: sequence-independent binding of the origin recognition complex. EMBO J 23: 191–201. 14685267 10.1038/sj.emboj.7600029 PMC1271667

[28] Papapetrou EP, Ziros PG, Micheva ID, Zoumbos NC, Athanassiadou A (2006) Gene transfer into human hematopoietic progenitor cells with an episomal vector carrying an S/MAR element. Gene Ther 13: 40–51. 16094410 10.1038/sj.gt.3302593

[29] Jackson DA, Juranek S, Lipps HJ (2006) Designing nonviral vectors for efficient gene transfer and long-term gene expression. Mol Ther 14: 613–626. 16784894 10.1016/j.ymthe.2006.03.026

[30] Lau WY, Lai EC (2008) Hepatocellular carcinoma: current management and recent advances. Hepatobiliary & pancreatic diseases international : HBPD INT 7: 237–257. 18522878

[31] Hidalgo M (2010) Pancreatic Cancer. New England Journal of Medicine 362: 1605–1617. 20427809 10.1056/NEJMra0901557

